# 2D nanomaterials for near infrared responsive photomedicine: a review

**DOI:** 10.1186/s40580-026-00577-7

**Published:** 2026-09-22

**Authors:** Su Bin Lee, Anara Molkenova, Seung Yun Yang, Ki Su Kim

**Affiliations:** 1 Department of Bioengineering, Hanyang University, Seoul, 04763 Republic of Korea; 2 Institute of Advanced Organic Materials, Pusan National University, Busan, 46241 Republic of Korea; 3 Department of Biomaterials Science, Life and Industry Convergence Institute, Pusan National University, Miryang, 50463 Republic of Korea

**Keywords:** Two-dimensional (2D) nanomaterials, Near infrared (NIR) laser, Photodynamic therapy (PDT), Photothermal therapy (PTT), Synergistic PDT/PTT, MXenes, 2D metal–organic frameworks (2D MOFs), 2D transition metal dichalcogenides (2D TMDs), 2D black phosphorus (BP)

## Abstract

The continuously growing demand for efficient and non-invasive treatment has accelerated the development of near infrared (NIR) responsive phototherapy, a therapeutic modality that leverages the biological transparent window (700–1850 nm) to enable deeper tissue penetration compared to ultraviolet (UV)/visible light approaches. NIR-responsive two-dimensional (2D) nanomaterials have emerged as particularly promising platforms for advanced photomedicine, attributed to high surface-to-volume ratio, tunable electronic structure, and broadband optical absorption. 2D nanomaterials can be broadly classified into elemental materials, layered nonmetal compounds, metal-based compounds, and 2D organic frameworks, each exhibiting distinct physicochemical and photonic features that enable tailored photothermal and photodynamic therapy applications. Their structural versatility and surface functionality further facilitate multimodal NIR theranostic integration. This review systematically investigates recent advances in NIR-responsive 2D nanomaterial-mediated phototherapy, with particular emphasis on their photomedical performance. Furthermore, the existing critical challenges, including biocompatibility, long-term biosafety, controlled degradation, NIR penetration depth limitations, and clinical translation barriers have been overviewed. Finally, future perspectives are proposed to guide the development of the next-generation NIR-responsive 2D nanomaterials for photomedicine.

**Graphical abstract:**

**Figure:**
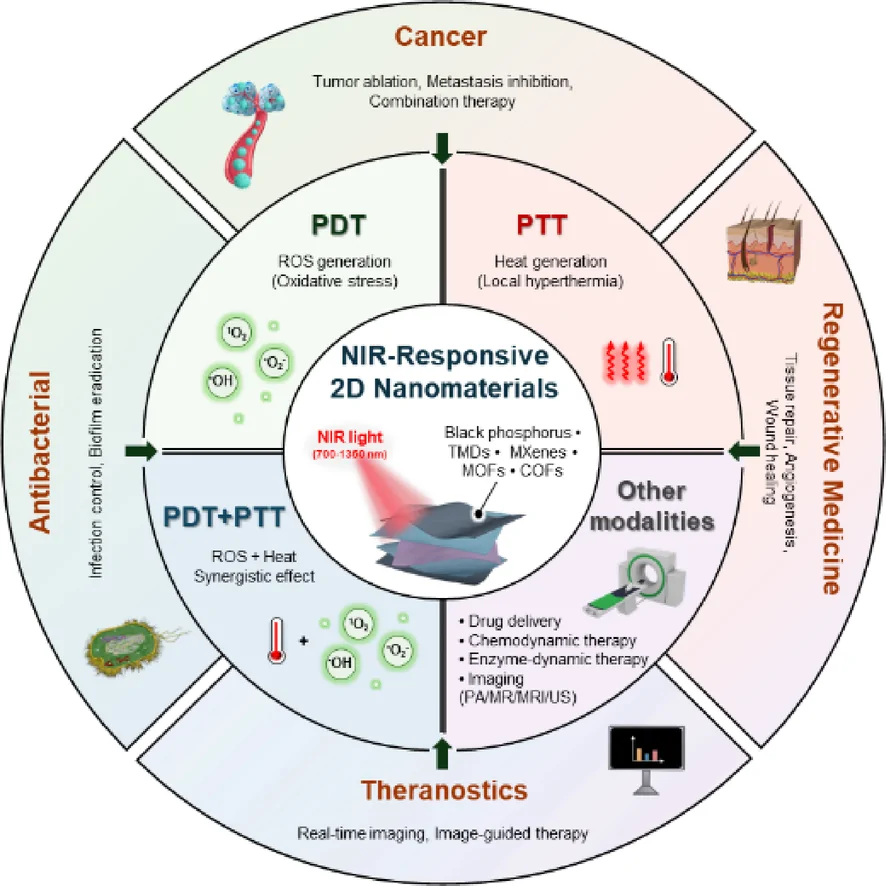

## Introduction

Photomedicine has emerged as a transformative paradigm in personalized medicine owing to its interdisciplinary approach to investigate and apply biological light-tissue interactions for the diagnosis, monitoring, and treatment of human diseases. Over the past years, phototherapy has been revolutionized by advancements in nanotechnology, which facilitates image-guided treatment strategies enabling the precise light-activated control of therapeutic agents and real-time monitoring of pathological tissues [1]. In principle, phototherapy utilizes photoresponsive agents to convert photons to therapeutic effects through various treatment mechanisms: (i) photothermal therapy (PTT), which leverages light-induced thermal ablation; (ii) photodynamic therapy (PDT), which employs photosensitizer to produce cytotoxic reactive oxygen species (ROS); (iii) photobiomodulation (PBM), which modulates cellular function to promote tissue regeneration; (iv) optogenetics, which modulates cellular function via genetic engineering; (v) phototriggered therapies, such as drug delivery and gas therapy [2–5].

While phototherapy modalities have conventionally relied on UV and visible light activation, these wavelengths are fundamentally constrained by poor tissue penetration capacity (< 5 mm) [6], due to high scattering coefficients and strong absorption by endogenous chromophores, such as hemoglobin and melanin. In this regard, NIR light (> 700 nm) has attracted attention in phototherapy due to its superior tissue penetration depths from millimeters to centimeters [7]. Biological tissues exhibit optical transparency windows with minimal scattering and absorption, such as NIR-I (760–1000 nm), NIR-II (1000–1350 nm), and NIR-III (1500–1800 nm). Hudson et al. [8] reported that 808 nm light (0.001W/cm^2^) penetrates 34 mm into bovine tissue (18–95 mm thick), which is 54% deeper than the 22 mm by 980 nm light (0.001 W/cm^2^). Typically, NIR irradiation across common therapeutic wavelengths (e.g., 808 nm ~1.54 eV, 980 nm ~1.27 eV, 1064 nm ~1.16 eV, 1567 nm ~0.79 eV) is capable of increasing the local tissue temperature by 10–20 °C without causing significant phototoxicity since its photon energy is beyond the DNA damage threshold (< 3 eV) [9]. While wavelength selection and penetration depth are crucial, the intrinsic light conversion properties of photoresponsive agents govern the therapeutic outcomes. In particular, the dimensionality of the material is a fundamental parameter for light matter interaction. Nanomaterials can be classified by their spatial confinement into zero-dimensional (0D, quantum dots, carbon dots), one dimensional (1D, nanowires, nanotubes), two dimensional (2D, nanosheets, nanoplatelets), and three dimensional (3D, mesoporous spheres) [10]. Among them, 2D nanomaterials, characterized by lateral dimensions in micrometer range and thickness confined to single (~ 1 nm) or few atomic layers (< 10 nm), have garnered unprecedented attention in phototherapy due to their unique properties: (i) ultrahigh surface-to-volume ratio that maximizes bioactive surface area and potential room for drug loading; (ii) tunable electronic properties enabling tailorable absorption bands; (iii) strong NIR absorption coefficients; (iv) efficient photothermal conversion efficiencies; and (v) intrinsic or sensitizer loadable ROS generation capabilities. These attributes 2D nanomaterials as versatile nanoplatforms for NIR-responsive phototherapy. Generally, 2D nanomaterials can be broadly classified into elemental materials (black phosphorus, BP [11, 12]; silicene [13]; boron [14, 15]), layered nonmetal compounds (graphitic carbon nitride, g-C_3_N_4_ [16]; hexagonal boron nitride [17]), 2D compounds (transition metal dichalcogenides, TMDs [18]; MXenes [19–22]; layered double hydroxides, LDHs [23]), and 2D organics (metal organic frameworks, MOFs [24, 25]; covalent organic frameworks, COFs [26]). Each class exhibits distinctive photophysical properties, which can be precisely modulated by adjusting chemical composition, layer number and surface functionalization, enabling tailored NIR-responsive photodynamic and photothermal applications [27].

This review systematically examines recent advances in NIR responsive 2D nanomaterials-mediated phototherapy beyond graphene. Given that graphene-based phototherapy has been extensively covered in literature, this review aims to highlight the latest developments in emerging 2D platforms [28]. We conducted a comprehensive literature analysis, compiling literature tables that categorize disease models (cancer therapy, antibacterial, regenerative medicine, etc.), multimodal integrations, including therapeutic modalities (PDT, PTT, synergistic PDT/PTT, etc.), and imaging techniques (photoacoustic, PA; magnetic resonance imaging, MRI; computed tomography, CT; ultrasound, US). Moreover, we extracted and tabulated laser settings (wavelength, power density, exposure period) to provide researchers experimental guidelines for designing their photoactivation protocols. Thus, this review is structured as follows: Sect. 2 briefly introduces 2D nanomaterial-mediated ROS dynamics and progress made in PDT pathways; Sect. 3 provides basics on 2D photothermal agent (PTA)-mediated heat generation mechanisms under NIR irradiation and discusses recent advances in PTT application; Sect. 4 analyzes synergistic PDT/PTT strategies and combination with other treatment modalities. Section 5 addresses translational challenges and suggests future perspectives of the next generation NIR-responsive 2D nanomaterials and artificial intelligence (AI) or machine learning (ML)-driven advances, and Sect. 6 concludes this review.

## Advances in 2D nanomaterial-based PDT

### Principles and mechanisms of 2D nanomaterials for PDT

**Fig. 1:**
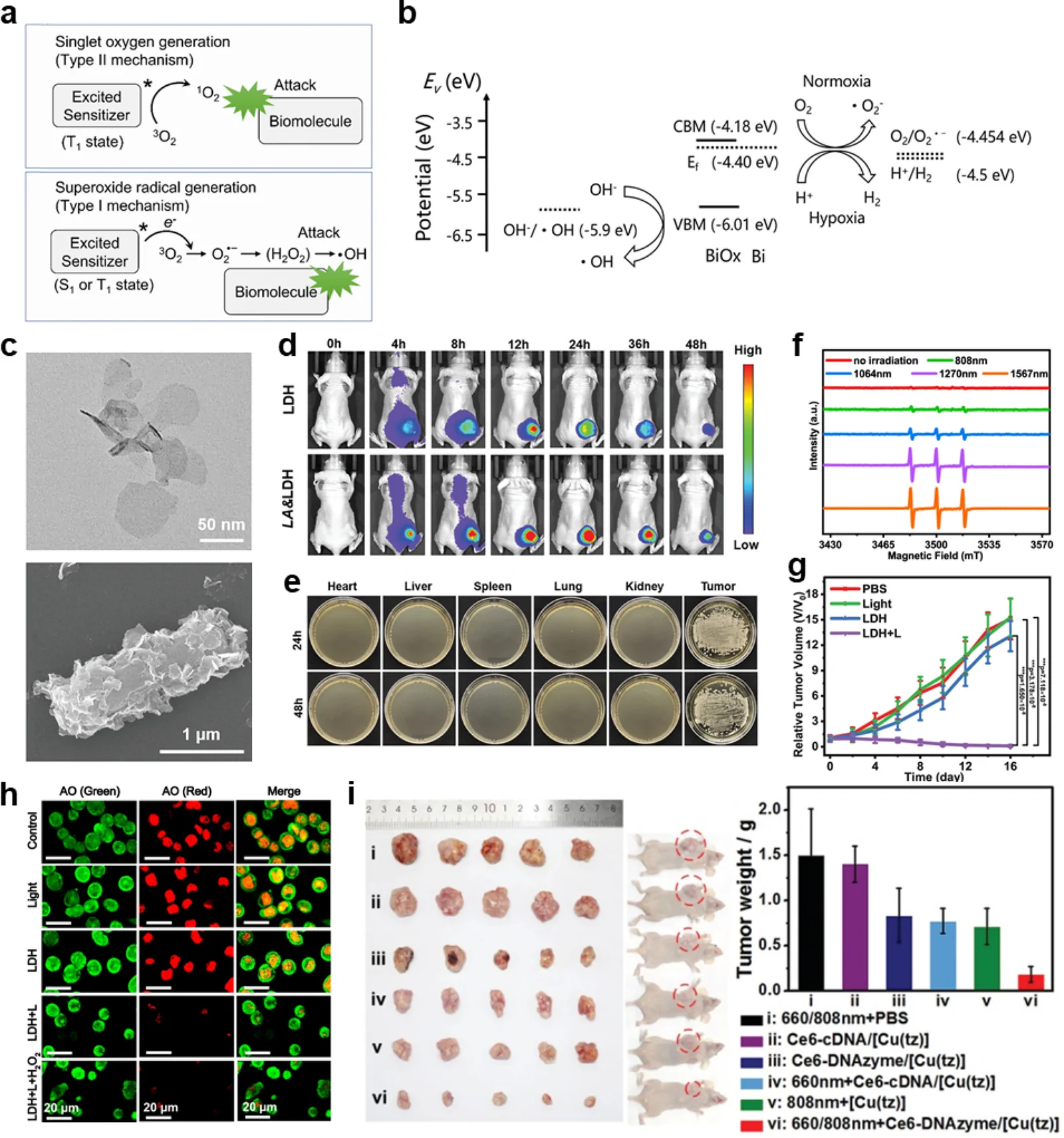
Mechanisms and applications of 2D nanomaterials-based PDT for cancer therapy. **a** Schematic representation of Type I and Type II mechanisms. Reproduced with permission from [42]. Copyright 2025 ROYAL SOCIETY OF CHEMISTRY. **b** Scheme of ROS generation principle of Bi/BiO_x_ nano heterostructure. Reproduced with permission from [46]. Copyright 2021 Wiley. **c** TEM images of CoCuMo-LDH and CoCuMo-LDH coated on LA. **d** NIR fluorescence imaging and **e** accumulation of LA&LDH for biodistribution analysis. Reproduced with permission from [48]. Copyright 2023 Wiley. **f** Electron spin resonance spectra for ^1^O_2_ detection under laser exposure (0.5 W/cm^2^) in various wavelength. **g** Tumor growth in different treatment groups with or without laser irradiation (1567 nm, 0.5 W/cm^2^). **h** AO staining for evaluating PDT-induced lysosome impairment. Reproduced with permission from [49]. Copyright 2022 Springer Nature. **i** Photographs of representative tumors and tumor weight after different treatments with or without laser irradiation (660 nm at 0.4 W/cm^2^, 808 nm at 0.6 W/cm^2^). Reproduced with permission from [50]. Copyright 2021 Wiley

PDT is an FDA-approved skin treatment method that can produce cytotoxic ROS exclusively upon light activation, destroying diseased tissue with minimal side effects. This ROS is mainly generated when a photosensitizing agent (PSA) is excited by a specific wavelength of light (commonly red light 630–650 nm), transferring energy or an electron to molecular oxygen or surrounding substrates. The resulting oxidative environment accelerates damage to essential biomolecules, specifically DNA, proteins, and lipids, facilitating efficient eradication of malignant cells or bacterial pathogens [29]. Recently, 2D nanomaterials have shown great prospects in expanding PDT therapeutic horizons by shifting photoactivation from conventional red light to deep tissue penetrating NIR. More importantly, 2D nanomaterials can serve as an active PSA or/and a carrier enabling them to build versatile PDT nanoplatforms with multimodal treatment capabilities. Similar to conventional PSAs, 2D nanomaterials can generate ROS through Type I and Type II mechanisms (Fig. 1a). Certain 2D semiconductor nanomaterials (e.g., C_60_ [30], MoS_2_ [31–33], MoSe_2_ [34, 35], Bi_2_Se_3_ [36], Bi_2_WO_6_ [37], WSe_2_ [38], SnTe [39], and MoO_x_ [40]) with engineered narrow band gaps tend to produce ROS via the Type I mechanism. Upon NIR photon absorption, photoexcited electrons migrate from valence band (VB) to conduction band (CB), forming electron–hole pairs capable of catalyzing redox reactions with O_2_, H_2_O, or H_2_O_2_ to produce ROS radicals, such as superoxide (O_2_·^−^) and hydroxyl radicals (OH·), even under the hypoxic tumor environment [41, 42]. For example, Kang et al. [43] reported Z-scheme heterojunction based on red phosphorus (RP) and BP exhibiting reduced energy loss. The photoexcited electrons recombined with holes in the VB of BP from the CB of RP, resulting in increased ROS production efficiency. Some 2D nanomaterials (e.g., Cu-TCPP MOF [44], LDH [45]) can reach a triplet state (T_1_) by absorbing NIR light, eventually transferring this energy to molecular oxygen (O_2_), converts triplet oxygen (^3^O_2_) to singlet oxygen (^1^O_2_) via Type II mechanism. For instance, Qiu et al. [46] demonstrated Bi/BiO_x_ nano heterostructure with suitable band alignment showed both Type I and Type II PDT (Fig. 1b). Photoactivated Bi transferred energy to ^3^O_2_ for ^1^O_2_ generation, as BiO_x_ induced ·OH by receiving electrons from Bi under both normoxia and hypoxia. In porphyrin-based MOFs, the intrinsic π-conjugated electron systems and additional nonbonding electrons enhance electron transfer and charge separation, thereby promoting efficient ^1^O_2_ generation, while Cu^2+^ centers induces a cascade of Fenton-like O_2_ production [47].

### 2D PDT nanoplatforms for cancer therapy

Since thin and flexible nanosheet structure can penetrate into the tumor tissue, 2D nanomaterials have been proposed as an ideal candidate for cancer treatment. In particular, up-regulation of cytotoxic ROS plays a key role in inducing cancer cell death due to the vulnerable redox balance of cancer cells. Although numerous treatments using the photodynamic effect of 2D nanomaterials activated by red light (e.g., 660 nm) have been widely studied [51, 52], this review highlights the efficacy of photodynamic applications using NIR light. For example, Yang et al. [48] developed CoCuMo-LDH nanosheets coated on the surface of *Lactobacillus acidophilus* (LA) for targeted accumulation in the tumor site (Fig. 1c). As shown in Fig. 1d and e, the presence of LA contributed to improved distribution within the tumor site and extended retention of LDH. CoCuMo-LDH nanosheets experience a loss of crystallinity under low pH and overexpressed glutathione (GSH). Notably, they employed NIR-II (1270 nm) to promote its ROS generation reaction. In another study, Shen et al. [49] utilized defect-rich CoMo-LDH nanosheets with polyethylene glycol (PEG) coating (DR-CoMo-LDH-PEG) under NIR-III (1567 nm) for deeper tissue penetration and highly efficient elimination of tumors. The electron spin resonance spectra of ^1^O_2_ (1:1:1) under different wavelength irradiation demonstrated 1567 nm could activate DR-CoMo-LDH-PEG with the highest performance (Fig. 1f). Accordingly, DR-CoMo-LDH-PEG with 1567 nm irradiation (0.5 W/cm^2^) group showed significant inhibition of tumor growth, as a result of lysosomal damage in tumor cells (Fig. 1g, h). Recent studies have also demonstrated that gene therapy, which reduces abnormally expressed genes of cancer, can be utilized to maximize therapeutic outcomes combined with PDT. For example, Liu et al. [50] reported Cu(I)-based coordination polymers (CP) nanosheets as an efficient carrier for Ce6-DNAzyme for catalyzed breakdown of target mRNA and Type II PDT, while Cu(I)-based CP nanosheets simultaneously promoted Type II PDT under 808 nm laser. As presented in Fig. 1i, this combined PDT and gene therapy exhibited notable tumor suppression. Notably, since NIR-responsive 2D nanomaterials exhibit high photothermal conversion efficacy, recent studies have predominantly reported synergistic effects with PTT rather than single PDT approaches. Thus, other applications are discussed in Sect. 4, which includes bone regeneration and wound healing.

## Advances in 2D nanomaterial-based PTT

### Principles and mechanisms of 2D nanomaterial-based PTAs

PTT is a minimally invasive therapeutic modality that employs light-to-heat converting PTAs to ablate tumor lesions or pathogens. When selecting a PTA, the photothermal conversion efficiency value (PCE or ${\eta}_{T}$, %) is a critical parameter, since light energy attenuates as it travels through the skin. Therefore, PTA must harvest the reached light to induce sufficient therapeutic hyperthermia. The PCR is commonly determined using the total energy balance method proposed by Roper et al. [53]. At steady state, heat generated by the plasmonic nanomaterial (${Q}_{I}$) and by background absorption of the sample cell (${Q}_{0}$) is balanced by heat dissipated to the surroundings:

1 $$hA\left({T}_{max}-{T}_{amb}\right)={Q}_{I}+{Q}_{0}$$

where *h* is the heat transfer coefficient; *A* is the surface area (cm^2^), ${T}_{max}$ is a steady state maximum temperature; and ${T}_{amb}$ is the ambient temperature. ${Q}_{I}=I(1-{10}^{-{A}_{\lambda }}){\eta}_{T}$ with *I* the incident laser power; ${A}_{\lambda }$ is the absorbance of 2D nanomaterial at the excitation wavelength (λ); ${\eta}_{T}$ is PCE. ${Q}_{0}$ is the heat dissipated due to background absorption by the sample cell/solvent, measured independently as ${Q}_{0}=\left(5.4\times {10}^{-4}\right)I \mathrm{J} {\mathrm{s}}^{-1}$ using a cell containing water without nanoparticles. Substituting the expression for ${Q}_{I}$ in Eq. 1 and rearranging for ${\eta}_{T}$ gives:

2 $${\eta}_{T}=\frac{hA\left({T}_{max}-{T}_{amb}\right)-{Q}_{0}}{I(1-{10}^{-{A}_{\lambda }})}$$

It should be noted that direct cross-literature comparison of PCE values among 2D materials is currently limited due to non-standardized experimental and thermodynamic modeling protocols. Reported PCE values vary drastically based on laser optics (excitation wavelength, beam size, power density, etc.), 2D material-dependent heat transfer dynamics (material concentration, layer number, or morphology-driven absorption, etc.), and experimental conditions (solvent heat capacity, cuvette geometry, heat dissipation assumptions, etc.) [54, 55].

**Fig. 2:**
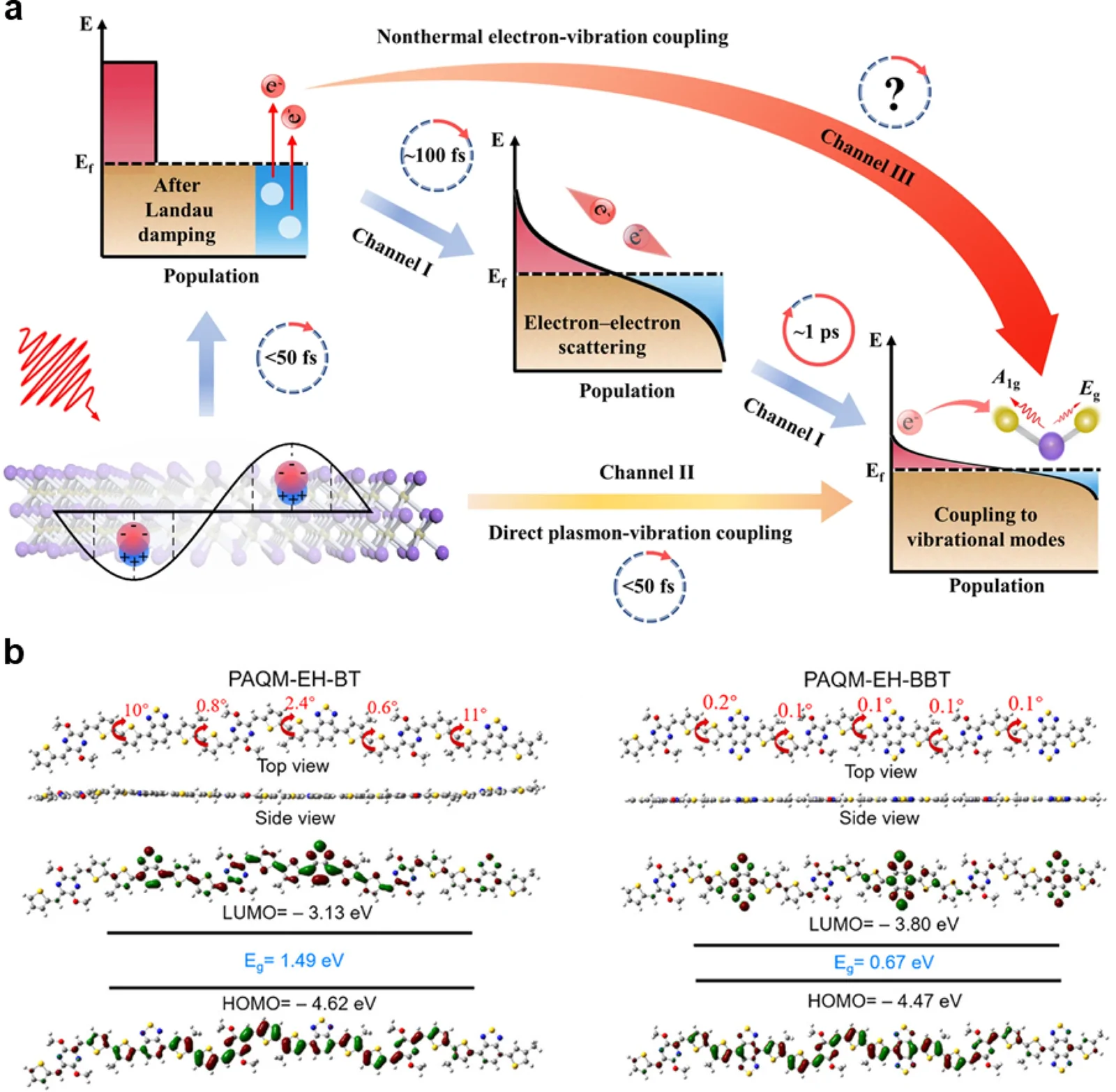
Photothermal mechanisms and structural strategies of 2D nanomaterials. **a** Conceptual schematic illustration of three surface plasmon (SP)-to-photon energy transfer pathways following optical excitation: Channel I, sequential relaxation via Landau damping, electron–electron scattering, and electron–phonon coupling; Channel II, direct SP-to-phonon coupling; Channel III, direct coupling of nonthermal electrons to phonons before electron–electron thermalization. This panel is purely schematic, irradiation wavelength and power density are not applicable. Reproduced with permission from [58]. Copyright 2022 Springer Nature. **b** Molecular designs and energy-level diagrams of NIR-II absorbing semiconducting polymers. The tailored acceptor strength, backbone planarity, and diradical character exhibit strong light harvesting ability in NIR-II window, enabling efficient PTT under 1064 nm (1.0 W/cm^2^). Reprinted with permission from [60]. Copyright 2025 Wiley

Exceptionally high PCE values of 2D PTAs have intensified the interest of researchers in PTT. For example, Li et al. [56] have determined that the internal photothermal conversion efficiency of Ti_3_C_2_T_x_ (T_x_ represents surface terminations, such as O, OH, and F) MXene is nearly 100%, which explains the reason why Ti_3_C_2_T_x_ is the most widely investigated PTA among other MXene analogs. In general, the photothermal conversion in metallic MXenes and other 2D plasmonics is driven by localized surface plasmon resonance (LSPR) [57]. As shown in Fig. 2a, when incident NIR light hits the plasmonic material, surface plasmons start to collectively oscillate and subsequently decay by transferring their energy to individual electrons evolving into “hot carriers” via a process called Landau damping [58]. These high-energy carriers then “relax” (cool down) by quick kinetic energy transfer towards the crystal lattice via electron–phonon interactions, enabling them to return to an equilibrium state. As a result, the generated thermal energy spreads out to the surrounding environment through a process governed by thermal boundary conductance (TBC) [59].

In contrast, the 2D semiconductor nanomaterials (e.g., MoS_2_ [61], GeP [62], MnO_2_ [63], etc.) also possess outstanding photothermal conversion properties, which mainly rely on non-radiative relaxation processes [64]. For example, Chen et al. [65] fabricated a 2D semiconductor tellurium-selenium (TeSe) based lateral heterojunction with an impressive PCE value of 99.1% under 808 nm irradiation, which enabled a near complete curative effect in preclinical models with human hepatocellular carcinoma and mouse lung cancer. In principle, the 2D semiconductors tend to absorb photons with energy exceeding their bandgap (E_g_), which excites electrons from the VB to the CB, producing mobile charge carriers in the form of electron–hole pairs. These excited carriers undergo non-radiative recombination via the Shockley–Read–Hall (SRH) or Auger process [66]. In SRH recombination, a carrier is trapped within deep-level impurities or bandgap defects, leading to electron–hole recombination. Alternatively, the Auger process involves three particle interactions, where energy from electron–hole recombination is transferred to the third carrier energy, which subsequently “cools” through scattering or lattice collisions. Eventually, the kinetic energy from both pathways is liberated into the crystal lattice via electron–phonon coupling, which converts electronic energy into vibrational energy (phonons), leading to a localized hyperthermia [66, 67].

Similarly, in specific 2D organics (e.g., periodic and layered π-π stacking structures), molecules excited by NIR light return from a higher excited singlet state to the ground singlet state primarily via nonradiative relaxation. Interestingly, NIR absorption of 2D organics tend to amplify upon aggregation, which is attributed to the stronger intermolecular electronic coupling and narrowed energy gap between highest occupied molecular orbital (HOMO) and lowest unoccupied molecular orbital (LUMO), inhibiting radiative relaxation in favor of heat generation [68]. However, several other complex mechanisms bestow 2D organics to generate PTT effect, including donor–acceptor interactions, radical stabilization, and structural confinement. In donor–acceptor interaction systems, the charge transfer endows strong NIR photon absorption. This electronic coupling can be modulated by strong donor and acceptor pairs to shrink the HOMO–LUMO energy gap and red shift the absorption toward the NIR-II window. For example, Zheng et al. showed that replacing conventional benzothiadiazole (BT) acceptor with the more electron-deficient benzobisthiadiazole (BBT) in para‑azaquinodimethane (p‑AQM)‑based quinoidal polymers lowers the LUMO and raises the HOMO, such narrowed gap afforded absorption band shift from the NIR-I to NIR-II (Fig. 2b) [60]. Furthermore, certain 2D organic structures can produce radicals via redox doping or photoinduction. These radicals are confined within 2D frameworks without degradation or quenching, facilitating broad NIR absorption and efficient photothermal conversion [69]. For example, Liu et al. [70] utilized Cairo pentagon tessellated 2D COFs with mcm topology to engineer a narrow bandgap (1.7–1.8 eV) and strong NIR absorbance (near 1000 nm). This unique low symmetry geometry with anisotropic electronic structure weakens the Coulombic attraction, facilitating efficient charge separation and nonradiative relaxation of electron–hole pairs, and thereby achieving a high PCE of 43.3%. Table 1 summarizes the recent designs and dominant limitations of 2D nanomaterials-based nanoplatforms for NIR-responsive single mode PDT or PTT applications.

**Table 1:** 2D nanoplatforms and key parameters for NIR-responsive single mode PTT/PDT in recent studies, including reported dominant limitations

2D nanoplatform		Disease model	Therapeutic/ imaging modalities	NIR laser settings			References	Dominant limitations
2D NSs	Other composition			*λ * (nm)	Power density (W/cm^2^)	Time (min)		
CoMo-LDH, NiMo-LDH	PEG	Breast cancer	PDT	1567	0.5	6	[49]	Biologically inert photosensitizers; poor ROS generation capability which highly depends on defect engineering
CoCuMo-LDH	Lactobacillus acidophilus	Breast cancer	PDT	1270	0.5	5	[48]	
Cu(I) 1,2,4-triazolate coordination polymer	DNAzyme, Chlorin e6	Breast cancer	PDT/Gene silencing	808	0.6	20	[50]	Intrinsic reliance on H_2_O_2_ levels
				660	0.4	10		
BP	Doxorubicin, Chitosan, Polyetheretherketone	Osteosarcoma/Bone regeneration	PTT/Chemotherapy	808	0.77	10	[77]	Rapid degradation and oxidation susceptibility of BP
TeSe	PVP	Hepatocellular carcinoma (HCC)/Lung cancer	PTT	808	1.0	10	[65]	Tumor-targeting mechanism unexplained; non-selective hyperthermia
CuFeTe_2_	PEG	Breast cancer	PTT/TME-response	1064	1.0	10	[72]	Multicomponent assembly with ratio dependence that hard to optimize
PtAg	Folic acid modified thoil-poly(ethylene glycol) (SH-PEG-FA)	Breast cancer	PTT/PA imaging	1064	3.25	10	[79]	Weak NIR-II extinction coefficient; high laser powers are needed; non-degradable; high cost
				785	1.5	10		
Pd–Pt	Ginger-derived extracellular vesicles,	Antibacterial	PTT/Electrodynamic therapy/PA imaging	980	0.5	20	[74]	Weak standalone PTT; Non-degradable; high cost
MoS_2_	S-nitrosothiol-modified chitosan	Antibacterial/Wound healing	PTT/Gas therapy	808	1.0	5	[129]	Poor photothermal stability in physiological environment
TiO_2_	CuS, HA, PEG	Atherosclerosis	PTT/SDT	1064	0.8	10	[76]	Wide bandgap with high charge-carrier recombination; non-degradable
CuFe_2_S_3_	PEG	Hepatocellular carcinoma	PTT/CDT	1064	1.0	5	[130]	TME-dependent CDT; short circulation half-life with liver spleen accumulation
Ti_3_C_2_T_x_ MXene	Paclitaxel, HA, PLGA	Melanoma	PTT/Chemotherapy	808	1.0	5	[78]	Poor stability in water and oxygen
Zr-Fc MOF	PEG, Carrageenan	Antibacterial/Wound healing	PTT/CDT	808	2.0	5	[25]	ROS output depends on pH and H_2_O_2_ levels
COF	DSPE-PEG-NH_2_	Breast cancer	PTT/ Pyroelectroimmunotherapy	808	1.0	10	[123]	O_2_-dependent ROS output; poor intrinsic dispersibility in water

(NSs, nanosheets; NIR, near-infrared; λ, wavelength; HA, Hyaluronic acid; PEG, Polyethylene glycol; PVP, Poly(vinylpyrrolidone); PLGA, Poly(lactic-co-glycolic acid); SDT, Sonodynamic therapy)

### 2D PTAs for cancer therapy

**Fig. 3:**
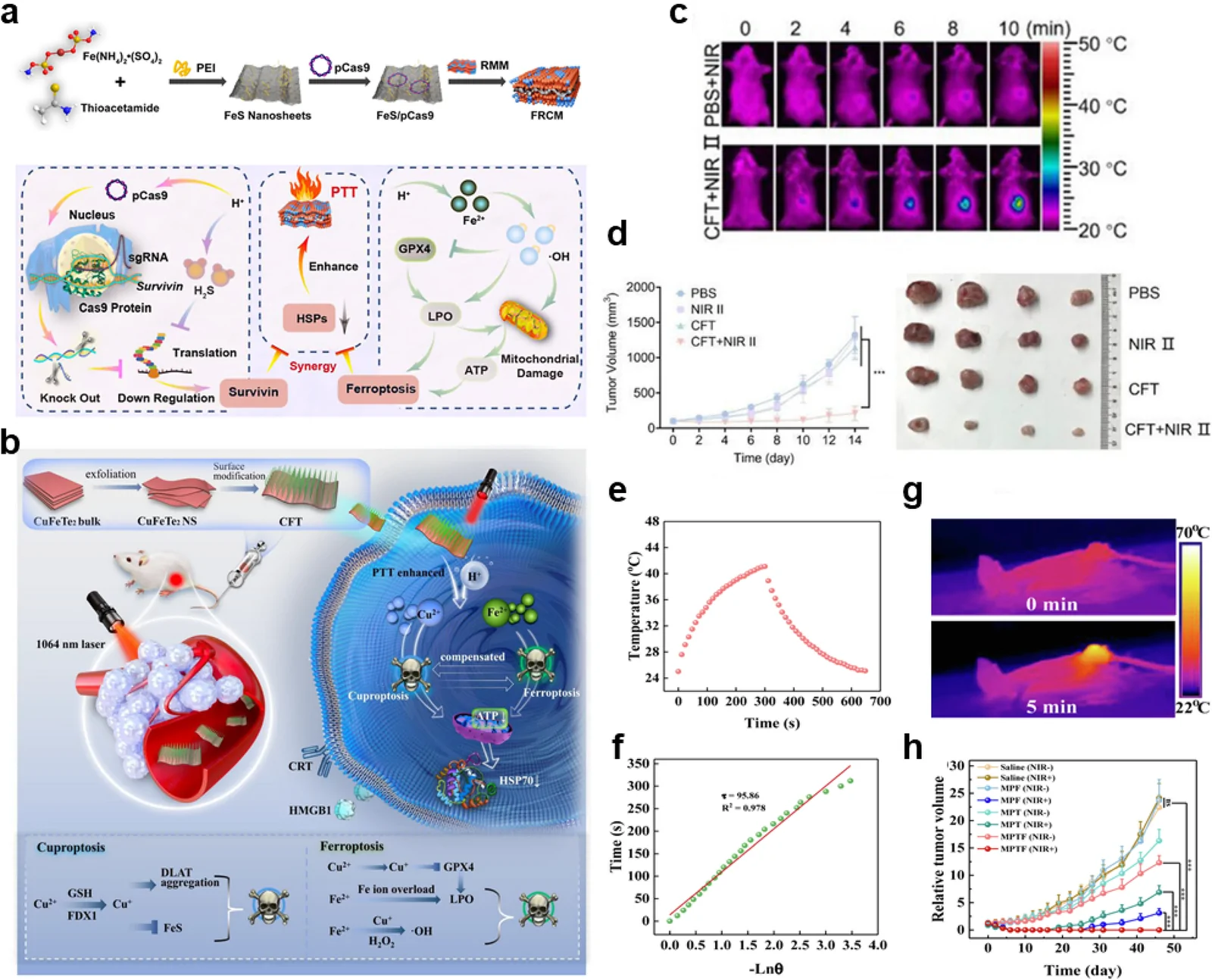
PTT of 2D nanoplatforms for cancer therapy. **a** Schematic representation of 2D FeS nanosheets synthesis integrated with CRISPER/Cas9 as PTA. Reproduced with permission from [83]. Copyright 2025 American Chemical Society. **b** Scheme of NIR-II PTT nanoplatform based on CFT. **c** Thermal images of tumor-bearing mice under 1064 nm laser irradiation (1.0 W/cm^2^). **d** Tumor volume growth curves and photographs on day 14 for different treatment groups. Reproduced with permission from [72]. Copyright 2025 Wiley. **e** Heating/cooling profiles and **f** cooling time versus -lnθ of 2D MoS_2_-based nanoplatform under NIR irradiation (808 nm, 0.3 W/cm^2^). **g** Thermal imaging of mice after 2D MoS_2_-based nanoplatform injection under NIR irradiation (808 nm, 0.3 W/cm^2^). **h** Tumor growth curves in different therapy groups. Reproduced with permission from [84]. Copyright 2022 Elsevier

PTT typically suppresses tumor growth via several synergistic mechanisms. More specifically, localized hyperthermia above 43 °C disrupts vital cellular functions by causing denaturation of essential proteins and enzymes [80]. Beyond this, the localized heat stress is also capable of damaging cell membrane integrity, leading to the leakage of intracellular components, and irregular tumor vasculature, leading to subsequent tumor deprivation in nutrients and oxygen. Depending on the heat intensity, tumor cells can undergo programmed (apoptosis) or unprogrammed (necrosis) cell death [81]. Yet, the PTT efficacy is challenged by the heat-shock proteins (HSPs), such as HSP70 and HSP90, which overproduction under heat-stress initiates a cellular defense mechanism that enables tumor cells to withstand hyperthermia [82]. To overcome photothermal resistance, Liu et al. [83] designed a bionic 2D iron sulfide (FeS) nanoplatform loaded with CRISPR/Cas9 (FCRM) (Fig. 3a). Under 808 nm NIR laser stimulation within acidic tumor microenvironment (TME), this nanoplatform decomposes by releasing Fe^2+^ ions and CRISPR/Cas9, which synergistically down regulate HSPs. Specifically, the Fe^2+^ ions catalyze lipid peroxidation, triggering ferroptosis of cancer cells additionally granting them MRI detectable contrast, while CRISPR/Cas9 well known as “scissors” of gene editing, reduces levels of anti-apoptotic protein Survivin to sensitize the cancer cells to photothermal damage . Recently, Feng et al. developed CuFeTe_2_ nanosheets (CFT)-based NIR-II PTT nanoplatform that releases Cu^2+^ ions and Fe^2+^ upon decomposition within TME (Fig. 3b). These free Cu^2+^ ions tend to reduce Cu^+^ by consuming intracellular GSH, thereby suppressing the activity of glutathione peroxidase 4 (GPX4). This inhibition, combined with Fe^2+^, amplifies lipid peroxidation (LPO) and triggers the synergistic cancer cell death mechanism by both ferroptosis and cuproptosis. Thermal imaging in Fig. 3c shows that CFT group tumors rapidly reach 43 °C under 1064 nm NIR-II irradiation (1.0 W/cm^2^). Consequently, the CFT+NIR II group exhibited considerable tumor growth inhibition (Fig. 3d), which, beyond thermal ablation, is also attributed to the induced immunogenic cell death and T-cell mediated immunity [72]. Biodegradable 2D nanomaterials, such as MXenes, often suffer from poor physiological stability, which significantly hampers their practical application. In a recent study, Lee et al. [78] addressed this issue by encapsulating Ti_3_C_2_T_x_ MXene nanoparticles within an amphiphilic polymer matrix composed of hyaluronic acid (HA) and poly(lactide-co-glycolide) (PLGA) to form HA-PLGA/MX NPs. Here, HA serves to target CD44 receptors overexpressed on the tumor cells, thereby improving tumor-specific cellular uptake. In addition, this nanoplatform leverages the high surface-to-volume ratio of MXenes to facilitate co-loading of paclitaxel (PTX), a potent antineoplastic chemotherapy drug that obstructs microtubules to block tumor cell replication. Consequently, under NIR laser irradiation HA-PLGA/MXP NPs showed a ∼95.7% tumor suppression rate in a melanoma mouse model.

A critical limitation of NIR-stimulated PTT is that laser power density applied on the skin must remain below the maximum permissible exposure (MPE) values for the specific wavelength and irradiation time. We calculated the MPE values according to the American National Standard for the Safe Use of Lasers (ANSI Z136.1) and laser-product safety requirements of IEC 60825-1 [85, 86]. The calculations assume continuous wave (CW) exposure of skin for durations of 10 s to 10^4^ s and a 3.5 mm diameter circular limiting aperture. For the wavelength range 400–1400 nm, the ANSI Z136.1 defines the skin MPE can be identified by 0.2 × *C*_A_ (W/cm^2^), where* C*_A_ is the wavelength-dependent correction factor, defined as follows: (i) *C*_A_=1.0 for CW exposure at 400–700 nm; (ii) *C*_A_=10^0.002(λ −700)^ (where λ is the laser wavelength in nanometers) for CW exposure at 700–1050 nm; (iii) *C*_A_=5.0 for CW exposure at 1050–1400 nm. Applying this relationship gives the MPE values, such as ~0.329 W/cm^2^ (808 nm), ~0.726 W/cm^2^ (980 nm), and ~1.0 W/cm^2^ (1064 nm). For the 1500–1800 nm wavelength range, IEC 60825-1 specifies a standardized skin MPE limit of 0.1 W/cm^2^ for exposure durations from 10 to 10^4^ s. Yet most studies employing 808 nm lasers exceed its safety threshold to achieve sufficient therapeutic outcomes. To address this issue, Li et al. [84] developed a PEGylated α‑tocopheryl succinate (α‑TOS) / folic‑acid‑conjugated 2D MoS₂ nanoflake platform (MoS₂‑PEG‑TOS‑FA or abbreviated as MPTF). The temperature of the MPTF solution was monitored under laser on (300 s) and laser off (cooling down) conditions (Fig. 3e). The cooling data was used to calculate τ_s_ versus –lnθ (Fig. 3f), from which a high PCE value of 65.3% was determined. Under safe 808 nm NIR-I irradiation (0.3 W/cm^2^), the MPTF groups showed the tumor temperature elevation up to 51.2 °C, sufficient to cause irreversible damage to cancer cells (Fig. 3g). Moreover, the covalently attached α‑TOS enables selective chemotherapy against cancer cells sparing normal ovarian cells, precluding infiltrating/metastatic cells and their further recurrence (Fig. 3h).

### 2D PTAs for regenerative medicine

#### Antibacterial bone regeneration

NIR driven mild PTT, which is typically conducted under < 45 °C, is a promising treatment approach for simultaneous treatment of bacterial infections and promoting bone regeneration. For example, Qu et al. [87] reported that Ti_3_C_2_T_x_ MXene-mediated mild hypothermia upregulated heat shock HSP70 protein expression, thereby activating the ERK signaling pathway to promote osteogenic differentiation of adipose-derived stem cells. Moreover, the 99% antibacterial efficiency was achieved against *Escherichia coli* (*E. coli*) and *Staphylococcus aureus* (*S. aureus*) and *methicillin-resistant Staphylococcus aureus* (*MRSA)* bacterial strains.

An emerging trend in regenerative medicine is NIR-driven pyroelectric dynamic therapy (PEDT), which provides spatiotemporal control over sterilization and tissue regrowth. When exposed to mild temperature fluctuations under NIR irradiation, polarized pyroelectric nanomaterials tend to generate charges that further react with oxygen and water in the vicinity, producing ROS. For instance, Qi et al. [88] designed an NIR-responsive Z-scheme pyroelectric nanoplatform through in situ growth of pyroelectric barium titanate (BaTiO_3_) nanoparticles onto sulfur-vacancy-rich S-MoS_2_ nanosheets with subsequent embedding into poly(L-lactic acid) (PLLA) matrix for the potential treatment of infected bone defects. Upon NIR exposure (808 nm, 1.0 W/cm^2^), S-MoS_2_ serves as a PTA that activates the pyroelectric effect of BaTiO_3_, electron–hole separation, and ROS generation for antibacterial inactivation of *E. coli* and *S. aureus*. In addition, ultrasonic stimulation was employed to induce the piezoelectric effect, which electrically stimulated osteoblast proliferation and differentiation, enabling simultaneous infection treatment and bone regeneration.

**Fig. 4:**
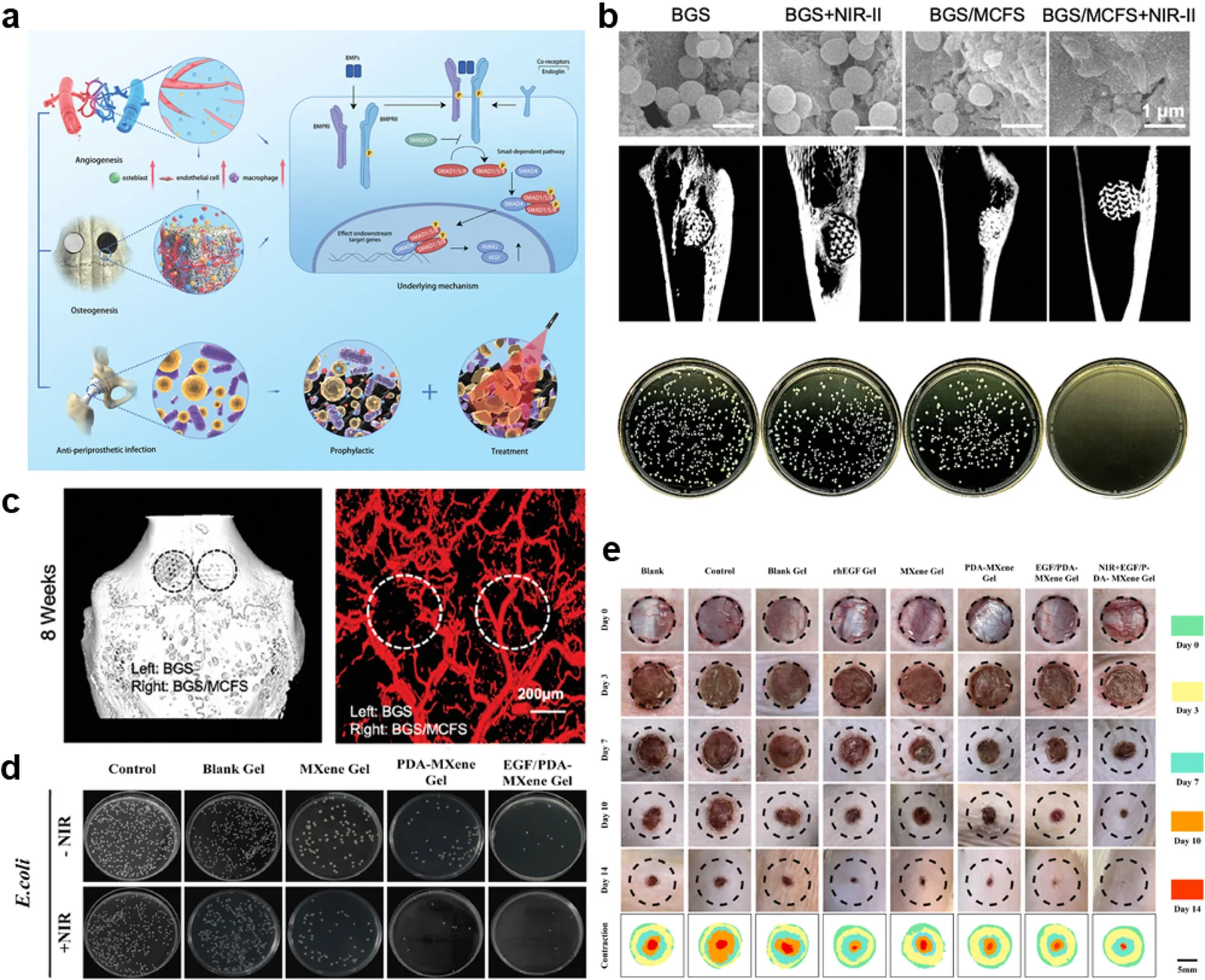
2D nanomaterials as a regenerative photomedicine. **a** Scheme of BGS/MCFS platform for bone regeneration and infection treatment. **b** SEM images of MRSA, micro-CT imaging of rabbit tibia and photographs of colony from muscle tissue after different treatments with or without laser irradiation (1064 nm, 1.0 W/cm^2^). **c** 3D micro-CT scan of bone formation and corresponding regenerated vessels induced by BGS and BGS/MCFS. Reproduced with permission from [23]. Copyright 2024 Wiley. **d** Antibacterial performance on *E.coli* and **e** Wound healing capability in different groups with or without laser irradiation (808 nm, 1.5 W/cm^2^). Reproduced with permission from [89]. Copyright 2025 Elsevier

Bian et al. [23] introduced 2D MgCuFe-LDH-derived polymetallic sulfide (MCFS) nanosheets into 3D-printed bioactive glass scaffold (BGS) to combat periprosthetic infections and vascularized osteogenesis under NIR-II light irradiation (Fig. 4a). In particular, the MCFS nanosheets are designed to release Mg^2+^, Cu^2+^ and Fe^3+^ ions that compromise bacterial vitality via suppressing critical physiological processes, such as energy production and nutrient metabolism (Fig. 4b). Moreover, the BGS/MCFS platform is capable of activating the transforming growth factor-β (TGF-β) signaling pathway, leading to an 8.5-fold increase in regenerated bone mass (Fig. 4c) and a 2.3-fold increase in neovascularization in a rabbit critical-size cranial defect model.

#### Wound healing

Miao et al. [89] designed EGF/PDA-MXene Gel for accelerated treatment of Type I diabetic wounds. In this system, MXenes serve both as a PTA and an epidermal growth factor (EGF) carrier, which allows it to exploit NIR laser as a remote drug release switch, ensuring a steady and continuous supply of EGF. Furthermore, the NIR-driven localized hyperthermia facilitates the bacteria eradication through protein denaturation (Fig. 4d). This photothermal effect subsequently promotes angiogenesis and restores microcirculation, which is essential for improving re-epithelialization and achieving closure of diabetic wounds by 99.14% after 14 days (Fig. 4e). Wang et al. [25] designed an NIR-responsive hydrogel platform via stabilizing zirconium-ferrocene MOF nanosheets within a polyethylene glycol dicarboxylic acid polymer network for potential application in antibacterial wound healing. The Zr-Fe MOF acts as a nanoenzyme sterilization platform capable of converting H_2_O_2_ into bactericidal ROS. Under 808 nm irradiation (2.0 W/cm^2^, 10 min), the localized heat disrupts bacteria and enhances the catalytic activity of Zr-Fe MOF, enabling the synergistic sterilization effect in the *S. aureus*-infected skin wound mouse model .

### 2D PTAs for antibacterial application

The crisis of antibiotic resistance poses a critical threat to global healthcare, necessitating urgent innovative solutions to combat recalcitrant bacterial infections. While PTT has shown great potential, electrodynamic therapy (often abbreviated as EDT) has emerged as a promising complementary strategy. Electrodynamic therapy employs electrosensitizers, such as metal alloys with profound electrocatalytic activity, that generate ROS under electric field stimulation. In a recent study, Qiao et al. [74] constructed a biomimetic therapeutic nanoplatform by conjugating 2D Pd–Pt alloy nanosheets onto the surface of ginger-derived extracellular vesicles (EVs) for synergistic antibacterial PTT/electrodynamic therapy. The biomimetic features of EVs enable the nanoplatform to internalize into bacteria cell interior in a lipid-dependent manner. This intracellular localization benefits the EV-Pd–Pt-assisted synergistic eradication of pathogens from within via NIR-driven hyperthermia and in situ electrocatalytic generation of ROS stimulated by a square-wave AC electric field.

### 2D PTAs for other applications

#### Atherosclerosis

PTT for atherosclerotic treatment has garnered considerable attention, owing to its ability to regulate plaque inflammatory macrophages through thermal ablation. Next-generation PTT nanoplatforms do not rely on hyperthermia alone, they are functionalized with additional treatment modalities to reprogram surviving macrophages. For example, Cao et al. [76] reported HA- and PEG-modified copper sulfide (CuS)/titanium dioxide (TiO_2_) heterostructure nanosheets (HNSs), which were capable of generating mild-hypothermia under 1064 nm NIR-II irradiation and ROS under ultrasound activation, subsequently inducing the proinflammatory macrophage apoptosis and inhibiting early atherosclerotic plaque progression.

#### Smart hydrogels

Unique photothermal conversion properties of PTA have gained considerable interest in the construction of photothermoelectric-responsive conductive hydrogels. Thermoelectric materials are capable of converting heat into electricity via the Seebeck effect [90]. For instance, Hou et al. [91] integrated 808 nm-photoresponsive Ti_3_C_2_T_x_ MXenes into a gellan-gum/poly (N-hydroxyethyl acrylate) polymer network. This synergistic design endows the hydrogel with outstanding mechanical strength, high conductivity, frost-resistance, and thermoelectric conversion characteristics. Consequently, such hydrogels open avenues for versatile applications, including human-motion and behavior monitoring, self-powered sensors, and real-time temperature feedback, and energy harvesting devices. In a recent study, Guo et al. [92] constructed a hand-shaped self-sensing actuator, which integrated m-MXene into a thermoresponsive poly(N-isopropylacrylamide) (PNIPAm) hydrogel with AI systems, demonstrating effective NIR-responsive bioinspired motion control. Sun et al. [93] designed an NIR-controlled hydrogel-based microvalve composed of anisotropic bilayer hydrogel (Laponite/PNIPAM) embedded with 2D-MoO_2_ nanosheets. When exposed to 808 nm NIR light (0.8 W/cm^2^), the hydrogel produces heat and undergoes volumetric shrinkage allowing the fluidic flow.

#### Bioimaging

Nibler et al. [94] engineered ultra-bright 2D copper tetrasilicate (MCuSi_4_O_10_, M = Ca, Sr, Ba) nanosheets with tunable near-infrared emission achieved through Group-II cations doping. In particular, CaCuSi_4_O_10_ nanosheets are widely known as Egyptian blue pigment. Under 808 nm excitation, the emission was successfully shifted from the conventional NIR-I region (~ 910 nm) into the NIR-II window (peaking at ~1007 nm). The resulting nanosheets exhibit a high photostability with a quantum yield up to 34%, enabling high-speed imaging at a frame rate above 200 fps. These materials facilitated superior high-resolution in vivo imaging, including transcranial cerebrovascular mapping and the first reported real-time tracking of single macrophages across murine cortex using diffuse optical localization imaging (DOLI), demonstrating their strong potential as next-generation NIR-II bioimaging contrast agents.

## 2D nanomaterials for synergistic PDT/PTT

### Synergistic PDT/PTT for cancer treatment

Due to their higher therapeutic efficacy, synergistic approaches of PDT and PTT have been increasingly employed for a wide range of applications and clinical trials. Mechanistically, PTT-induced mild hyperthermia (41–45 °C) promotes the performance of PDT by increasing intratumoral blood flow to enhance O_2_ supply essential for ROS generation. Simultaneously, the elevated thermal energy destabilizes cancer cell membranes by increasing lipid bilayer fluidity, which enhances the cellular uptake and accumulation of PTA. On a macromolecular level, the combined heat stress and oxidative shock induce severe denaturation of proteins and deactivate critical HSPs and DNA repair enzymes thereby disrupting cellular recovery process and rendering cancer cells susceptible to apoptosis and necrosis [95]. Furthermore, synergistic PTT/PDT triggers a cascade of immunogenic cell death (ICD). Mechanistically, this dual-modality stress induces the critical translocation and surface exposure of calreticulin (ecto-CRT), which acts as a powerful “eat-me” signal for phagocytes [96]. This exposure occurs alongside the active secretion of adenosine triphosphate (ATP) and the extracellular release of high mobility group box 1 (HMGB1) and intracellular HSPs. Collectively, these damage-associated molecular patterns (DAMPS) promote the recruitment and maturation of dendritic cells. Mature dendritic cells facilitate tumor antigen and present them to T cells, stimulating generation of interferon gamma. As a result, the host immune system is thoroughly activated by inducing the proliferation and infiltration of cytotoxic T lymphocytes (CD8+T cells), to eradicate both primary tumors and distant metastases [97]. In a recent study, Zhao et al. [98] highlighted violet phosphorus (VP) nanosheets, which were stable in the biological environment compared to BP, to induce PDT, PTT, and catalytic therapeutic outcomes. Moreover, in the past few years, PDT and PTT combined with other therapeutic modalities such as chemotherapy [99] or mitochondrial membrane potential damage (MMPD) [100] have been extensively investigated.

#### 2D PDT/PTT combined chemodynamic therapy

**Fig. 5:**
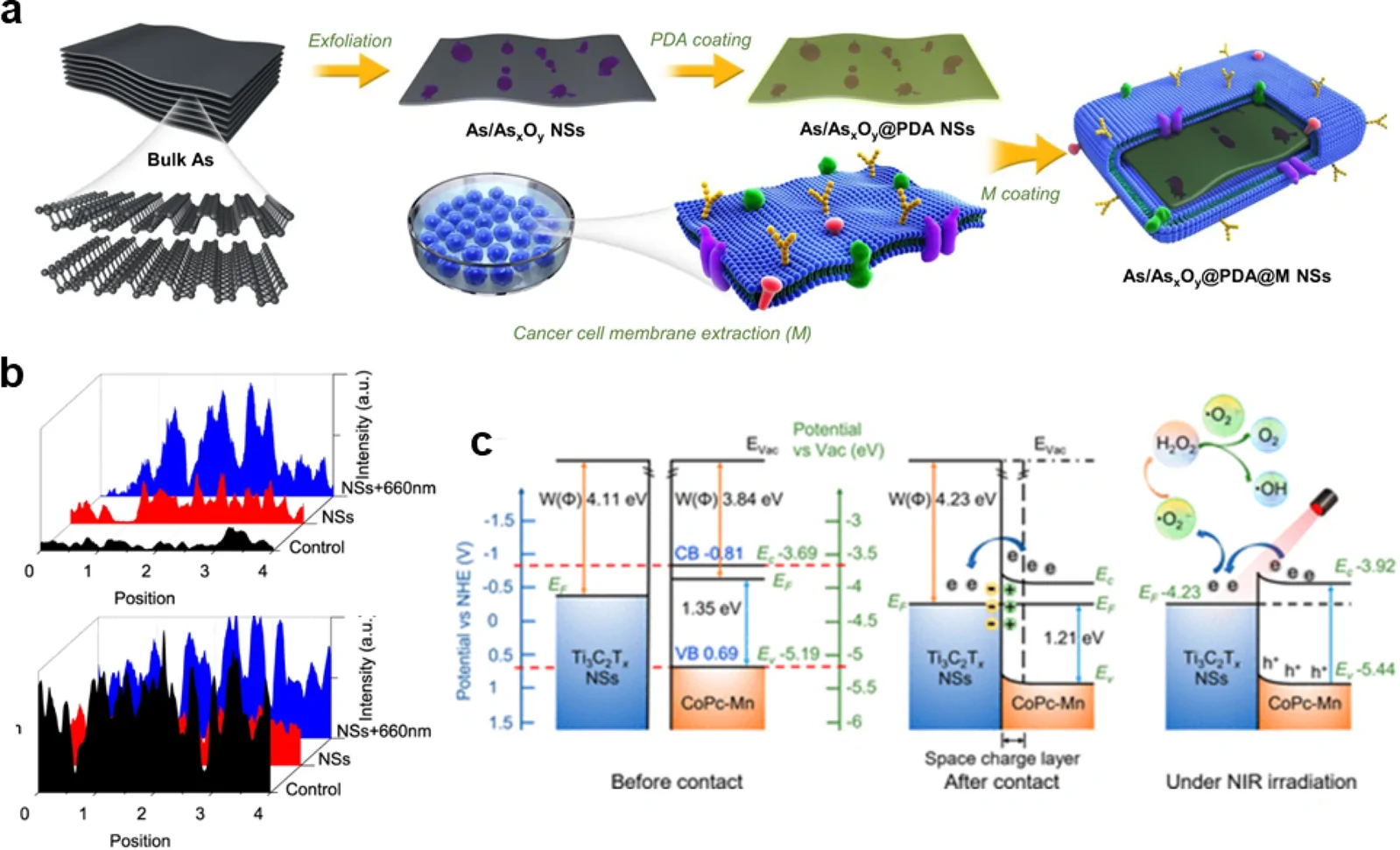
CDT combined synergistic PDT/PTT for cancer therapy. **a** Schematic illustration of the fabrication of an As/As_x_O_y_ nanosheet-based PDT/PTT platform combined with CDT. **b** Fluorescence quantitative analysis of intracellular ROS and O_2_ levels of As/As_x_O_y_@PDA@M NSs under 660 nm laser (0.3 W/cm^2^). Reproduced with permission from [105]. Copyright 2021 Springer Nature. **c** Schematic illustration of the changes in the energy band alignment of Ti_3_C_2_T_x_ and CoPc-Mn nanosheets before contact, after contact, and under irradiation (808 nm, 1W/cm^2^), demonstrating the proposed mechanism of NIR-responsive ROS generation. Reproduced with permission from [106]. Copyright 2023 American Chemical Society

Chemodynamic therapy (CDT) has been considered as an emerging therapeutic strategy, which initiates Fenton or Fenton-like catalytic reactions to produce highly toxic ·OH radicals within disease microenvironment [101, 102]. However, the Fenton reaction rates are relatively slow, which can be resolved by coupling with PTT [103]. Sun et al. [104] reported that Fenton kinetics was fourfold augmented when the local temperature was elevated from 20 to 50 °C. Moreover, in situ oxygen evolution capabilities of CDT offer great prospects for hypoxia sensitive PDT. This synergistic O_2_ and ROS generation can sensitize tumor cells by depleting their antioxidant defense through GSH neutralization. Thus, the development of versatile 2D nanoplatforms for synergistic CDT/PDT/PTT can offer significant clinical benefits by overcoming inherent limitations of each modality. For instance, Kong et al. [105] developed an intelligent theranostic platform based on arsenene nanosheet (As/As_x_O_y_) with the Type II heterojunction with longer electron–hole pair lifetime suitable to catalyze ·OH and O_2_ from H_2_O_2_ and prevent ROS consumption by GSH. Notably, arsenic trioxide (As_2_O_3_) is now FDA-approved for the acute promyelocytic leukemia treatment. The cancer cell membrane encapsulation of nanosheets with partial surface oxidation facilitated enhanced active targeting ability and extended their blood circulation (Fig. 5a). Also, As/As_x_O_y_ nanosheets demonstrated excellent photothermal conversion performance under 808 nm laser (2.0 W/cm^2^, 5 min) and in vivo fluorescence image guidance. Moreover, in situ self-generation of O_2_ alleviates hypoxic TME, maximizing the PDT efficacy under 660 nm irradiation (Fig. 5b) .

The Schottky junction engineering strategy is widely employed to overcome the bottleneck of charge dynamics in 2D nanomaterials [107–109]. For example, Duan et al. [106] investigated Schottky junctions established between a metal layer (Ti_3_C_2_T_x_ MXenes) and a semiconductor (CoPc-Mn nanosheets) that integrates Type I and II photosensitizer properties for PA imaging-guided synergistic PDT/PTT/CDT. When Ti_3_C_2_T_x_ MXenes with metallic nature come into close contact with CoPc-Mn nanosheets, charge redistribution triggers Fermi levels equilibration and interfacial band bending, forming the localized potential barrier (Schottky barrier). This barrier can act as an electron trap by channeling electrons exclusively towards MXenes and confining holes within the semiconductor, thereby minimizing charge recombination and maximizing their utilization for ROS production upon 808 nm irradiation, as shown in Fig. 5c. Notably, NIR laser irradiation triggers thermionic emission by elevating local temperature, which imparts electrons thermal kinetic energy to escape from the Schottky barrier into the semiconductor. This process further enhances the charge separation, ultimately boosting the ROS yield for synergistic PDT/PTT/CDT [109].

#### 2D PDT/PTT combined enzyme dynamic therapy

**Fig. 6:**
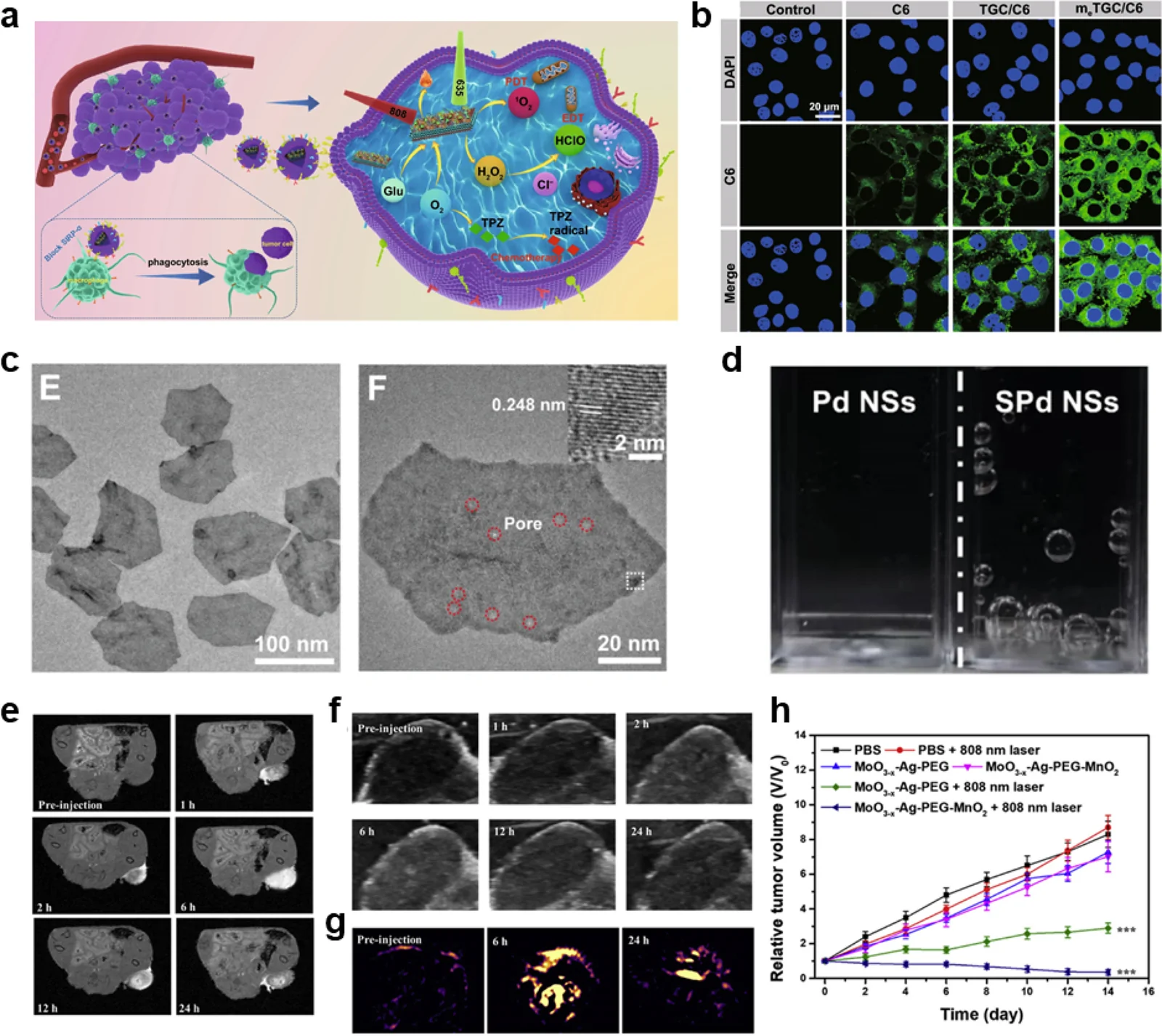
Other modalities combined synergistic PDT/PTT for cancer therapy. **a** Schematic illustration of Ti_3_C_2_ MXene-based PDT/PTT integrated with EDT and chemotherapy in cancer. **b** Enhanced intracellular uptake for cancer cell membrane coated nanosheets. Reproduced with permission from [110]. Copyright 2022 Springer Nature. **c** TEM images of SPd NSs. **d** Photographs of O_2_ bubbles produced by CAT-like activity of SPD NSs. Reproduced with permission from [111]. Copyright 2022 Wiley. **e** MRI, **f** US, **g** PA images of tumor model mice treated by MoO_3-x_-based nanoplatform. **h** Tumor growth monitoring in different treatment groups with or without laser irradiation (808 nm, 1.2 W/cm^2^). Reproduced with permission from [112]. Copyright 2021 Elsevier

Enzyme dynamic therapy (EDT) utilizes a cascade of natural or inorganic enzymes to produce ROS and O_2_ within TME, which makes it a powerful synergistic tool to mitigate hypoxia in PDT, while PTT can benefit by accelerating enzyme-catalyzed reactions. For example, Zhang et al. [110] designed a bionic cascaded-enzyme nanoreactor by conjugating glucose oxidase (GOx) and chloroperoxidase (CPO) enzymes onto Ti_3_C_2_ MXene nanosheets, which was further loaded with hypoxia-sensitive tirapazamine (TPZ) drug and camouflaged with gene-engineered cancer cell-membrane with over-expressed CD47 (m_e_TGCT) (Fig. 6a). Importantly, over-expressed CD47 endow “don’t eat me” signal, enabling immune evasion, long blood circulation, and efficient tumor cell uptake through homologous self-recognition (Fig. 6b). Once accumulated at the tumor site, the m_e_TGCT nanoplatform can simultaneously produce heat and ROS under 635 nm/808 nm dual laser activation. Specifically, MXene-mediated hyperthermia promotes enzymatic activities, where GOx catalyzes glucose oxidation into H_2_O_2_, which is consumed to form hypochlorous acid (HClO) by CPO. Under hypoxic conditions, TPZ is converted into cytotoxic benzotriazine (BTZ) radical, which traps topoisomerase II enzyme on DNA, subsequently inducing the double strand break.

#### 2D PDT/PTT combined nanozyme-based catalytic therapy

Nanozyme-based catalytic therapy employs nano-engineered materials that mimic natural enzyme activities to modulate redox balance within TME, either by generating or scavenging ROS. Li et al. [111] introduced lattice tensile strain into palladium nanosheets (SPd NSs) to activate inactive facets by surface reconstruction (Fig. 6c). Such lattice “stretching” lifts the d-band center closer to the Fermi level, endowing stronger adsorption of the reactant molecules. As a result, these SPd NSs exhibited higher NIR-driven photocatalytic activity (up to 2-fold), such as catalase (CAT) and peroxidase (POD)-like activity without combining other metals (e.g., gold and platinum) or organic photosensitizers. Figure 6d shows O_2_ generation by SPd NSs from H_2_O_2_ induced by CAT-like activity, while POD-like activity produces ROS.

#### 2D cancer theranostics

Due to their unique physical properties, 2D nanosheets can integrate multiple diagnostic capabilities along with synergistic treatment modalities. For example, Wu et al. [112] developed MRI, PA, US, and fluorescence imaging-guided phototherapy nanoplatform, composed of Ag nanocubes, MoO_3-x_ nanosheets, and MnO_2_ nanoparticles (MG-MoO_3-x_-Ag-PEG-MnO_2_). In acidic TME conditions, MnO_2_ undergoes reaction with endogenous H_2_O_2_ in the presence of H^+^ ions yielding O_2_ gas, Mn^2+^ ions, and water molecules. The released Mn^2+^ ions produce significant bright contrast on T_1_-weighted MRI (Fig. 6e), while O_2_ microbubbles are capable of generating distinct acoustic contrast in US imaging (Fig. 6f). Meanwhile, Ag/MoO_3-x_ composite converts absorbed NIR photonic energy into heat, producing strong thermoelastic expansion generating ultrasound waves detectable by PA imaging (Fig. 6g) [113]. Apart from imaging, superior PDT/PTT therapeutic effect was observed by significant tumor growth inhibition shown in Fig. 6h**,** underscoring nanoplatform’s tremendous potential for cancer theranostics.

### 2D PDT/PTT for bone regeneration

**Fig. 7:**
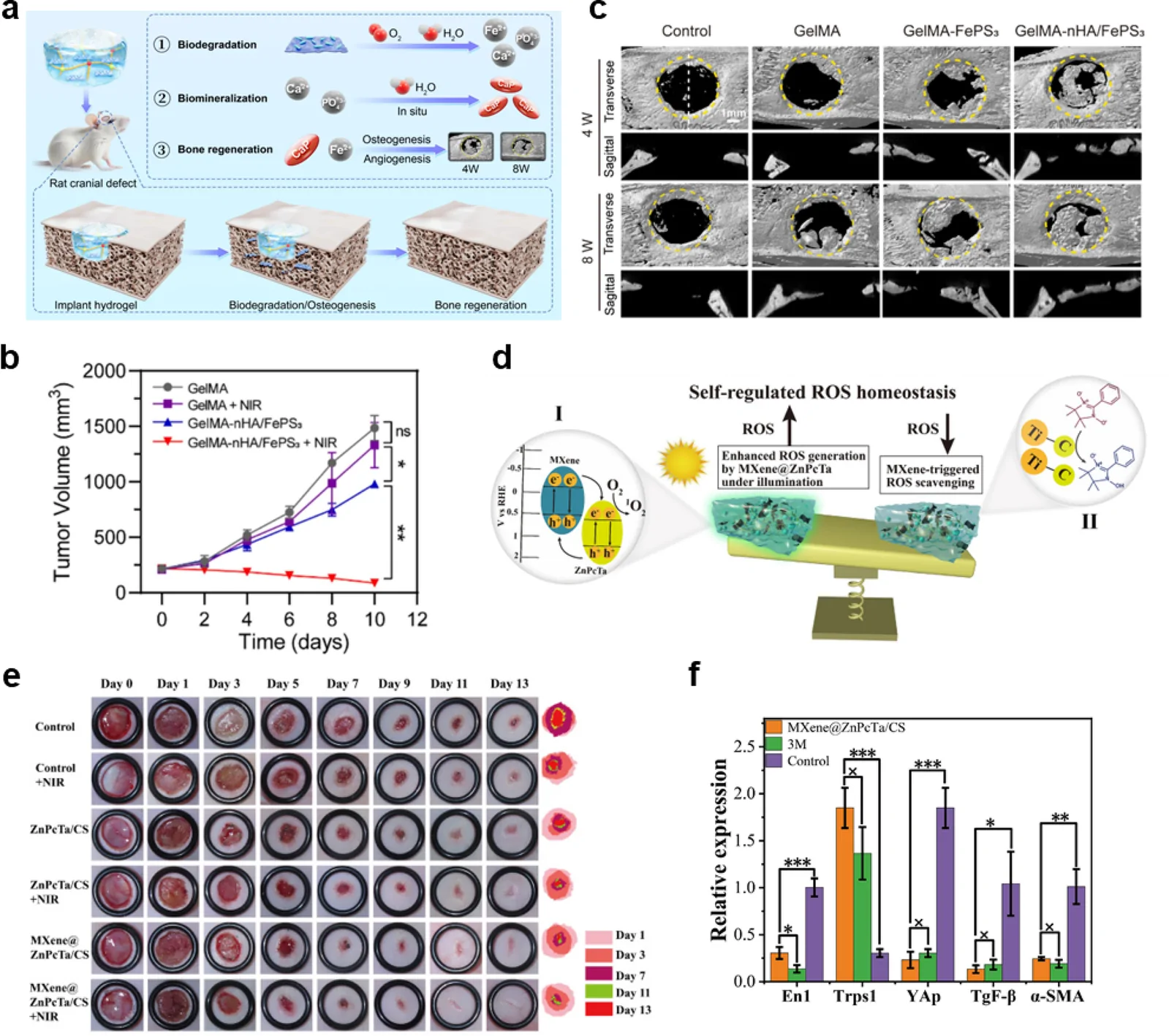
Synergistic PDT/PTT for tissue regeneration. **a** Schematic representation of therapeutic strategy for osteosarcoma and bone repair platform. **b** Relative tumor volume in different treatment groups with or without laser irradiation (808 nm, 1.0 W/cm^2^). **c** Micro-CT imaging of calvarial defect site after various treatments. Reproduced with permission from [114]. Copyright 2025 Elsevier. **d** Schematic illustration of ROS regulation system of MXene@ZnPcTz/CS gel-based dressing. **e** Wound healing progress in various treatment groups with or without laser irradiation (808 nm, 0.5 W/cm^2^). **f** mRNA expression related to tissue-retentive properties in rat skin after different treatment. Reproduced with permission from [116]. Copyright 2025 Elsevier

Beyond their roles in cancer treatment, 2D nanomaterials have been explored in bone regenerative medicine. In particular, phosphorus-based 2D materials garnered significant attention owing to their ability for in situ phosphorus-driven biomineralization. For instance, Ye et al. [114] synthesized multifunctional nanohydroxyapatite (nHA) loaded FePS_3_ nanosheets incorporated in gelatin methacrylate (GelMA) hydrogel (GelMA-nHA/FePS_3_) for treating both osteosarcoma and bone repair (Fig. 7a). Photothermal and photodynamic therapeutic effects were triggered by FePS_3_ nanosheets under 808 nm NIR irradiation. The resulting hyperthermia accelerates dynamic redox cycling Fe(II)/Fe(III) in Fenton reactions of FePS_3_ leading to depletion of intracellular GSH. Thus, without supply of GSH, GPX4 is unable to maintain cellular redox homeostasis triggering ferroptosis-dependent tumor cells death (Fig. 7b**)**. Furthermore, FePS_3_ eventually degrades releasing phosphate ions, while nHA provides sustained release of calcium ions, promoting osteogenic differentiation. In micro-CT images, GelMA-nHA/FePS_3_ scaffold promoted remarkable bone regeneration (Fig. 7c). Pan et al. [115] also proposed BP nanosheets (BPNs) into thermoresponsive chitosan (CS) hydrogel combined with platelet rich plasma (PRP) for rheumatoid arthritis treatment. This BPNs/CS/PRP hydrogel promoted removal of persistent hyperplastic synoviocyte and abnormal tissues by PDT and PTT under 808 nm (1.0 W/cm^2^) irradiation.

### 2D PDT/PTT for infected wound healing

Extensive overuse of antibiotics has created an explosive growth in global prevalence of multidrug resistant bacteria. By virtue of their ultrathin and faceted structure with atomically sharp edges, 2D nanomaterials have shown enormous potential to mechanically cut bacterial cell membranes, while the NIR driven heat and ROS simultaneously inactivate the multidrug resistant bacteria. For instance, Zeng et al. [116] presented multifunctional wound dressing by combining MXene and ZnPcTa into the CS hydrogel. This MXene@ZnPcTz/CS gel-based dressing provided antibacterial therapy and angiogenesis via synergistic PDT/PTT, alleviating oxidative stress and inflammation from redox activity of MXene (Fig. 7d). Additionally, the mechanical contraction of the hydrogel promoted the wound closure by reducing its tension (Fig. 7e). The mRNA expression levels related to mechano-transduction properties from wound tissue demonstrated the self-healing and scar inhibition ability of MXene@ZnPcTz/CS (Fig. 7f). In another work, Li et al. [117] prepared a single-component Pd nanosheet by combining photothermal and photodynamic effects, while further enzyme-like activity contributed to chemical sterilization. Notably, they compared gene expressions in mouse wound tissue after treatment by RNA sequencing. This demonstrated up-regulation of genes related to skin development (e.g., EGF and KGF) and angiogenesis (e.g., FGF-2 and VEGF-A), whereas down-regulation of genes involved in pro-inflammatory pathways (e.g., TNF-α and IL-6) indicating resolved inflammation.

In the antibacterial literature, ROS-mediated bacterial sterilization by semiconducting nanomaterials is termed as an antibacterial photocatalytic therapy (APCT) [118, 119]. For example, Mo et al. [120] integrated oxygen vacancy rich cobalt-doped Bi_2_O_2_S (Co-BOS) nanosheets into thermosensitive self-shrinking hydrogel composed of chondroitin sulfate and Pluronic F127. Upon 808 nm NIR exposure, Co-BOS induces both photocatalytic ROS generation and photothermal effect, showing effective antimicrobial and wound closure in MRSA biofilm-infected mouse wound model.

In conclusion, Table 2 summarizes the recent designs of 2D nanoplatforms for NIR-responsive synergistic PDT/PTT applications. It is evident that no single 2D nanomaterials can meet the clinical criteria for all disease types. MXenes offer strong broadband NIR absorption suited for combined PTT/PDT/CDT platforms, but rapid oxidation, stacking/aggregation, and high charge-carrier recombination limit their long-term performance and reproducibility [106, 110, 121]. 2D organic frameworks, such as COF and MOF, offer an alternative design paradigm in which pore architecture and metal node chemistry can be rationally tuned to embed photosensitize or catalytic sites. However, the therapeutic performance is often directly tied to the amount of the loaded photosensitizer, and these platforms face the following limitations, such as hypoxia sensitivity, poor aqueous dispersibility for COF and low heat/ROS-generation efficacy alongside potential toxicity risks, such as metal ion leaching for MOF [122, 123]. Mono-elemental 2D materials, such as BP and Sb, present favorable biodegradability profiles, making them ideal for “transient” therapeutic platforms where long-term tissue persistence is undesirable [124]. However, the same susceptibility to physiological degradation compromises PTT/PDT efficacy, and batch-to-batch reproducibility, and in the case of Sb, raises concerns over the toxicity of degradation by products [125, 126]. In particular, BP transforms into biocompatible phosphate upon physiological oxidation, which can be strategically utilized for applications requiring localized biomineralization, such as bone regeneration [127]. However, this inherent susceptibility to oxidation introduces a clinical trade-off, because rapid degradation can interfere with light absorption, compromise photothermal efficacy, and reduce batch-to-batch structural reproducibility. Transition metal 2D compounds, such as MoO_3-x_ and Bi_2_S_3_, offer complementary imaging modalities (MR/PA/US/CT) and broad absorption, but limitations include defect-driven instability, pH-dependent degradation and rapid photo-induced electron–hole recombination [112, 128]. Across 2D nanoplatforms, hypoxia sensitive or oxygen-dependent ROS generation emerges as recurring constraint for PDT or CDT efficacy, underscoring the need for oxygen-independent or hypoxia-tolerant photosensitizer designs. The lack of long-term (> 12 months) clearance data and toxicological profiles of their degradation byproducts are common limitation across all the tabulated 2D nanomaterials, so we omitted this statement to avoid repetition. Ultimately, the rational material selection and optimization should be tailored to the specific therapeutic requirements.

**Table 2:** 2D nanoplatforms and key parameters for NIR-responsive synergistic phototherapy in recent studies, including reported dominant limitations

2D nanoplatform		Disease model	Therapeutic/imaging modalities	NIR laser settings			References	Dominant limitations
2D NSs	Other composition			*λ *(nm)	Power density (W/cm^2^)	Time (min)		
BP	Fe_3_O_4_@MnO_2_, NaYF_4_:Yb/Er/Nd, poly(acrylic acid), polylysine, Chlorin e6	Cervical cancer	PTT/PDT/MR/US/FL imaging	808	2.0	5	[131]	O_2_-dependent ROS output; rapid degradation and oxidation susceptibility
	Chitosan, Platelet rich plasma	Rheumatoid arthritis	PTT/PDT	808	1.0	8	[115]	
VP	–	Breast cancer	PTT/PDT	808	1.0	5	[98]	O_2_-dependent ROS output; poor physiological stability, short shelf-life, scalability issues
Pd	PVP	Antibacterial, wound healing	PTT/PDT	808	1.5	3	[117]	Non-degradable; retention in liver and spleen; PDT/PTT trade-off; scalability issues; high cost
	PVP	Breast cancer	PTT/PDT/PA imaging/Catalytic therapy	808	1.5	10	[111]	
Sb	PEG, 5,10,15,20-Tetrakis(4-hydroxy-phenyl)− 21H,12H-porphine (THPP)	Breast cancer	PTT/PDT/PA/FL imaging	808	1.0	10	[132]	O_2_-dependent ROS output; rapid physiological degradation; toxicity of degradation by products
				660	0.5	5		
As/As_x_O_y_	Polydopamine, Cancer cell membrane	Breast cancer	PTT/PDT/CDT/Fluorescence Imaging	808	1.0	10	[105]	Systemic toxicity; rapid physiological degradation; rapid clearance; scaling up issues
				660	0.3	10		
Ti_3_C_2_T_x_ MXene	CoPc-Mn, PEG, AS1411	Melanoma	PTT/PDT/CDT/PA imaging	808	1.0	10	[106]	Rapid oxidation; stacking and aggregation; high charge-carrier recombination; lack of intrinsic active targeting
	GOX, CPO, Tirapazamine, CD47-overexpressed cancer cell membrane	Breast cancer	PTT/PDT/Chemotherapy/Enzyme dynamic therapy	808	1.5	2	[110]	
				635	0.5	5		
	ZnPcTa, Chitosan	Antibacterial, wound healing	PTT/PDT	808	0.5	10	[116]	
Bi_2_S_3_/Ti_3_C_2_	PEG, Triphenylphosphium bromide	Glioblastoma	PTT/PDT/CT imaging	808	1.0	10	[128]	Narrow bandgap; O_2_-dependent ROS output; rapid photo-induced electron–hole recombination;
MoO_3-x_	Ag, PEG, MnO_2_	Cervical cancer	PTT/PDT/MR /PA /US imaging	808	1.2	5	[112]	Inherent instability of defects; O_2_-dependent ROS output; pH-dependent degradation
FePS_3_	Nanohydroxyapatite, Gelatin methacrylate	Osteosarcoma, bone regeneration	PTT/PDT/CDT	808	1.0	6	[114]	pH dependent CDT; complex degradation synchronization with bone growth; systemic toxicity of released ions
BiO_x_Cl	PEG	Breast cancer	PTT/PDT/CT/PA imaging	1064	1.0	5	[133]	Wide bandgap; instability of oxygen vacancies; rapid photo-induced electron–hole recombination;
Cu-THQ MOF	Au NPs	Antibacterial, wound healing	PTT/PDT	808	1.7	10	[122]	Low PCE and ROS generation capacity; Cu^2+^ ion release and potential toxicity

(NSs, nanosheets; NPs, nanoparticle; NIR, near-infrared; λ, wavelength; GOX, glucose oxidase; CPO, chloroperoxidase; PEG, Polyethylene glycol; FL, Fluorescence)

## Clinical translation outlook and future perspectives

Herein, we have reviewed research advances in NIR-responsive 2D nanomaterials, which represent one of the most active frontiers in contemporary photomedicine. 2D nanomaterials offer intrinsically superior surface-to-volume ratios, anisotropic charge, phonon transport, and thickness- and defect-tunable electronic structures. These unique features afford broadband and strong red-shifted NIR absorbance, exceptionally high PCE values, abundant surface sites for drug loading, and facile incorporation into hydrogels/scaffolds/microneedle platforms. Consequently, 2D-based therapeutic nanoplatforms have displayed remarkable performance in single mode and synergistic PDT/PTT, while simultaneously enabling combination with chemotherapy, chemodynamic therapy, gas therapy, and immunotherapy across diverse disease models, such as solid tumors, antibiotic-resistant infections, rheumatoid arthritis, periodontitis, atherosclerotic plaques, and chronic wounds. Despite these impressive advances, several critical challenges must be systematically addressed before 2D photomedicines can achieve successful clinical translation. The following key bottlenecks and future opportunities we would like to highlight:

### NIR laser safety

The clinical translation of 2D PDT/PTT is primarily constrained by the skin MPE limits for NIR laser irradiation, especially for deep-seated or poorly vascularized disease lesions. Tables 1 and 2 summarize the laser parameters reported in the analyzed literature. Many studies using 808 nm employed power densities ranging from 0.5 to 2.0 W/cm^2^, which substantially exceed the corresponding MPE of ~0.329 W/cm^2^ derived in Sect. 3.2, and thereby presenting a major barrier to clinical translation. Shifting to the NIR-II (1000–1350 nm) relaxes this constraint, with the skin MPE reaching 1.0 W/cm^2^ above 1050 nm, and most NIR-II studies in Tables 1 and 2 operated within or near that limit, while maintaining effective therapeutic outcomes. Remarkably, further extension into NIR-III (1550–1870 nm) is more demanding with an MPE of only 0.10 W/cm^2^. At such low power densities, a therapeutic temperature can only be achieved with materials of high PCE and strong molar extinction coefficient. Future 2D therapeutic nanoplatforms should therefore be optimized for high therapeutic efficacy under photoactivation within the safe exposure limits.

### On-demand activation

The “ON/OFF” or “on demand” activation is critical for precise spatiotemporal control and avoiding collateral damage. Some 2D LDH, MOF, and COF nanosheets can be tailored for controlled disassembly in response to pathological stimuli, such as pH 5.5–6.5, elevated levels of GSH 2–10 mM, H_2_O_2_ 50–100 μM, or upregulated enzymatic activities such as matrix metalloproteinases. Such a “turn on” feature enables PDT and/or PTT treatment activation exclusively within diseased tissue, leaving circulating nanosheets inactive. Furthermore, smart surface functionalization, including CS, PEG, zwitterionic polymer shells, etc., can be designed for pH-triggered charge change, enzyme-catalyzed degradation, or redox-responsive bond collapse. These dynamic transformations improve tumor accumulation by maintaining colloidal stability and immune evasion. Notably, biomimetic camouflaging (leukocyte, cancer cells, platelet, red blood cells, or extracellular vesicles-mimetic layers) are particularly promising for elegant suppression of immune recognition. Selected 2D nanosheets, containing manganese oxide, iron oxide, or copper catalytic centers, can in situ convert TME H_2_O_2_ into oxygen to relieve tumor hypoxia and enhance Fenton-like reactions, thereby avoiding systemic oxidative toxicity. In addition, macroscale NIR-responsive carriers, for example, sprayable black phosphorus gels [134] and nanostructured chitosan-polyphenolic patches [135], offer a parallel translational strategy. These carriers keep the therapeutic material at the lesion site and allow on-demand NIR-triggered activation, with the released dose controlled by irradiation time. Thus, we expect that future 2D nanoplatforms will be routinely designed for smart control through chemical, physical, and optical triggers, to achieve sufficient therapeutic precision and safety profiles suitable for clinical translation.

### Biodistribution, metabolism and clearance of 2D nanomaterials

Perhaps the most critical concern of 2D nanomaterials is their long-term in vivo fate, degradation kinetics, and potential for chronic organ toxicity. Most 2D nanosheets possess thicknesses of 1–100 nm and lateral dimensions of 50–500 nm, which are beyond the glomerular filtration threshold (< 6 nm), unless fragmented they are prone to accumulate in liver, spleen, lungs, and lymphoid organs [136]. Hou et al. [137] reported that flake-like 2D geometry of BP nanosheets (BPNs) promote preferential kidney accumulation in both healthy and acute kidney injury (AKI) mice, an effect attributed to their topographical similarity to rectangular DNA origami frameworks. This passive renal accumulation together with antioxidative properties of BPNs were employed to scavenge elevated ROS levels in injured kidneys of AKI mice. Biodistribution of Cy5-labeled BPNs was evaluated by in vivo imaging. In AKI mice, about 60% of BPNs were concentrated in kidneys among main organs (heart, liver, spleen, lung, and kidney) at 3 h post-injection, increasing to 80% at 12 h. However, a stability test of physisorbed Cy5 label revealed ~26% release after 12 h, suggesting that the latter value may reflect the location of the dye rather than intact BPNs. These values represent relative imaging-based biodistribution data rather than absolute dose fractions, and therefore cannot be used for accurate and quantitative comparison with other studies. Moreover, as this observation was made in a glycerol-induced AKI model, in which renal clearance is pathologically suppressed, this biodistribution pattern should not be generalized to healthy animals without independent validation.

Certain 2D nanomaterials, such as TMDs (MoS_2_, WSe_2_, etc.) and MXenes, can undergo in vivo fragmentation and dissolution. Lin et al. [139] reported that poly(vinylpyrrolidone) (PVP)-coated Nb_2_C MXene nanosheets are susceptible to enzymatic degradation by human myeloperoxidase (hMPO), with subsequent gradual excretion. The blood circulation half-time (t_1/2_) was 1.31 h, and nearly 20% of Nb was eliminated via urine and feces by 48 h post-injection. Biodistribution, quantified by inductively coupled plasma optical emission spectrometry (ICP-OES), revealed passive intratumoral accumulation of 2.24% ID/g. Similarly, hydrogen-terminated germanene nanosheets (GeH-PVP) undergo rapid oxidative degradation in oxygen-containing aqueous media, the suspension becoming translucent within 24 h, and X-ray photoelectron spectroscopy confirmed conversion of the Ge–Ge framework to Ge–OH and GeO_2_. For comparison, BP, Ti_3_C_2_ Mxene, and graphene oxide exposed to identical conditions retained their 2D morphology unaffected. The long-term in vivo evaluation of GeH-PVP over 4 weeks, including hematology, blood biochemistry, and histology of major organs, revealed negligible systemic toxicity across doses of 5–20 mg/kg [138].

Clearance kinetics of 2D materials are not an intrinsic property but are also affected by local environment (pH, H_2_O_2_, enzyme, etc.) and NIR irradiation protocols. For example, Fu et al. [71] reported that NIR irradiation accelerated the degradation and fragmentation of a MoS_2_/HfO_2_-dextran (M/H–D) nanoplatform. Inductively coupled plasma mass spectrometry (ICP-MS) quantification revealed that NIR irradiation increased the 10-day degradation ratio from 66.1 to 72.9% in simulated body fluid and from 75.9 to 90.1% in simulated lysosomal fluid, completely collapsing into ~5 nm particles by day 10. Despite the translational potential of these biodegradable 2D nanomaterials, their complete metabolic fate remains largely unresolved. Systematic long-term in vivo tracking and clearance assessments of their biodegradation products are therefore essential to define clinical applicability of these emerging nanomaterials.

### AI-assisted 2D nanomaterial engineering

AI and ML are expected to revolutionize the design of 2D nanoplatforms by accelerating the exploration of versatile new compositions, structures, and mechanical properties. Recent studies have demonstrated that ML models trained on density functional theory (DFT) datasets can accurately predict bandgap, PCE values, degradation kinetics and biological interaction (cellular uptake, immunogenicity, biodistribution, etc.) [73, 75]. AI-driven inverse design frameworks can simultaneously optimize multiple simultaneous parameters (2D thickness, composition, defect density, mechanical flexibility) and ML-based optimization shows prospects to navigate through identifying optimal properties (stiffness, bending modulus, lateral size, etc.) for therapeutic efficacy. Furthermore, employing DFT calculation and quantitative modeling of light penetration, absorption cross-section, thermal diffusion, and in vivo fluence distribution should be integrated with the ML algorithm would enable rational design to pre-screen 2D nanomaterial candidates and NIR irradiation protocols, reducing reliance on empirical trial-and-error optimization and accelerating clinical adoption.

## Conclusions

This review provided a comprehensive overview of NIR-responsive 2D nanomaterials beyond graphene for phototherapy applications, highlighting their principles and mechanisms as PDT and PTT agents. Owing to their effective photoreactive characteristics, 2D nanomaterials have demonstrated superior performance for single-mode and combined PDT/PTT across a broad range of biomedical applications, such as cancer treatment, antimicrobial therapy, tissue regeneration, cardiovascular disease treatment, and diagnostic imaging. In addition, the synergistic integration with other complementary therapeutic modalities (e.g., photo-driven drug release, chemodynamic therapy, electrodynamic therapy, gene therapy, and enzyme dynamic therapy) along with imaging modalities (e.g., CT, MRI, PAI, and FI) have further highlighted their theranostic potential. Despite this exciting progress, there are still several challenges that remain to be addressed. In particular, next generation 2D nanoplatforms should provide sufficient therapeutic efficacy at clinically relevant and thermally safe NIR doses. Surface engineering and integration with polymers, hydrogels, biomembranes, or bioactive scaffolds should be evaluated not only for dispersibility and targeting, but also to enable predictable biodistribution, biodegradation, clearance, and long-term safety. Continued research and development of novel 2D nanomaterials, supported by ML and AI, may accelerate the discovery of new structures with improved imaging and therapeutic capabilities, and disease-selective photoactivation. As these issues are resolved, 2D nanomaterials show bright prospects for advancing future phototherapeutic and diagnostic applications in nanomedicine.

## Data Availability

All data supporting the findings of this review are derived from previously published literature, which are cited throughout the manuscript. Summary tables and comparative analyses are included in the article.
